# Proteomic profiling of arteriovenous fistula tissue identifies dysregulated oxidoreductase proteins in diabetic end-stage renal disease

**DOI:** 10.3389/fendo.2026.1583065

**Published:** 2026-02-02

**Authors:** Bin Zhao, Shen Zhan, Xue Zhou, Pei Yu, Yuzhu Wang

**Affiliations:** 1 Department of Nephrology, Beijing Haidian Hospital, Beijing, China; 2 Department of Nephrology, Tianjin Haihe Hospital, Tianjin, China; 3 Department of Nephrology, Chu Hsien-I Memorial Hospital, Tianjin Medical University, Tianjin, China; 4 Department of Nephrology, The Second Hospital of Tianjin Medical University, Tianjin, China

**Keywords:** end-stage renal disease (ESRD), oxidoreductases, potential biomarker, proteomics analysis, type 2 diabetes mellitus (T2DM)

## Abstract

**Background:**

Diabetes mellitus is a leading cause of end-stage renal disease (ESRD), with up to 35% of patients with diabetes mellitus developing kidney disease. This study aims to monitor protein expression changes in ESRD patients with and without type 2 diabetes mellitus (T2DM).

**Methods:**

A total of 186 ESRD patients who underwent arteriovenous fistula creation surgery were enrolled in this study. Of these, 148 patients were classified into the T2DM (n = 73) and non-T2DM (n = 75) groups. Data-independent acquisition proteomic analysis was conducted to analyze differentially expressed proteins. Enzyme-linked immunosorbent assay kits, immunohistochemical staining, Western blotting were employed to validate the differently expressed proteins within the cohort.

**Results:**

Proteomic analysis identified 26 upregulated and 15 downregulated proteins in the T2DM group compared with the non-T2DM group. The serum concentrations of 4-hydroxynonenal, malondialdehyde, and glutathione were elevated in the T2DM group. The immunohistochemical staining index for GPX4 and xCT was lower, while α-SMA, IL-6, TNF-α, and TGF-β levels were higher in the T2DM group compared with the non-T2DM group. Western blotting indicated the downregulation of SOD1 and GPX4 as well as upregulation of PTGS2 and ACLS4 in the T2DM group accompanied by increased levels of Fe^2+^, total iron, and Fe^3+^.

**Conclusion:**

This study underscores oxidoreductase activity-related proteins, including ferroptosis-related proteins to be differentially expressed in ESRD combined with T2DM.

## 1 Introduction

End-stage renal disease (ESRD) is the final stage of kidney disease, caused by various risk factors (1). Recently, the prevalence of ESRD has significantly increased due to population ageing (2). Type 2 diabetes mellitus (T2DM) is a leading cause of ESRD and contributes substantially to cardiovascular diseases (3). In the United States, diabetes mellitus accounts for 30–50% of incident ESRD cases, and up to 35% of patients with diabetes mellitus develop kidney disease (4). According to a global atlas, the global annual incidence of ESRD among diabetic patients increased from 375.8 to 1016.0 per million population between 2000 and 2015 (5).

Although hemodialysis (HD) affects glucose homeostasis and insulin pharmacokinetics, it remains the primary management approach for ESRD patients combined with T2DM (6, 7). Currently, American and European vascular access guidelines recommend the use of arteriovenous fistulas (AVFs) as the optimal choice for vascular access in patients undergoing HD (8). Patients benefit from AVF use, as it significantly reduces morbidity, hospitalization costs, infection risks, and the incidence of thrombotic complications (9). However, AVFs have a major limitation, with an approximately 25–60% primary maturation failure rate (10), and diabetes mellitus is recognized as a risk factor for AVF failure (9). A deeper understanding of ESRD pathogenesis in combination with T2DM could improve HD treatment outcomes for patients with diabetic kidney disease.

Proteomic technologies are widely applied in clinical settings to facilitate early detection, diagnosis, prognosis, and monitoring of disease progression or therapeutic response (11). These technologies are effective in identifying differentially expressed proteins, providing invaluable and clinically relevant insights. For instance, Chen et al. (12) conducted aqueous humor proteomic analysis and identified 54 differentially expressed proteins enriched in cell adhesion and extracellular exosome pathways in patients with diabetic kidney disease. Zhao et al. (13) performed a transcriptomic-proteomic analysis using glomerular samples from patients with diabetic kidney disease, suggesting that inflammation-related proteins may contribute to disease progression. Proteomic technologies may offer a broad-based, unbiased scientific approach for understanding the complex nature of diabetic kidney disease (14).

The aim of this study was to characterize differentially expressed proteins in AVF samples collected from ESRD patients with or without T2DM. To achieve this, 186 ESRD patients were enrolled, and data-independent acquisition (DIA) proteomic analysis along with immunohistochemical staining were conducted. This study is the first to reveal differentially expressed proteins associated with oxidoreductase activity in AVF tissues collected from patients under diabetic kidney disease conditions.

## 2 Materials and methods

### 2.1 Study design and patients

This is a prospective observational study including ESRD patients who underwent AVF surgery at Beijing Haidian hospital between April and December 2024. The inclusion criteria were as follows: (1) individuals had to have a diagnosis of stage 5 ESRD, (2) participants had to be 18 years of age or older with an expected clinical survival of over six months, (3) and participants had to be undergoing AVF surgery for the first time, with surgery performed by end-to-side anastomosis of the cephalic vein (end) to the radial artery (side), involving less than one-third of the forearm as the surgical area. Additionally, (4) successful maturation of the AVF had to be achieved without the use of assistive strategies. Patients were excluded if they had initiated peritoneal dialysis or HD, had severe comorbidities, experienced declining renal function, or had plans to undergo kidney transplantation or switch to peritoneal dialysis. This study was approved by the Beijing Haidian Hospital Medical Ethics Committee (approval number: M202396). Written informed consent was obtained from all hospitalized patients. Patients were classified as either T2DM or non-T2DM based on their T2DM status.

Diabetes was defined by the presence of any of the following criteria: 1) glycated hemoglobin (HbA1c) ≥ 6.5%; 2) fasting plasma glucose (FPG) ≥ 7.0 mmol/L; 3) 2-hour plasma glucose level during a 75-g oral glucose tolerance test (OGTT) ≥ 11.1 mmol/L; or 4) random plasma glucose ≥ 11.1 mmol/L in a patient with classic symptoms of hyperglycemia or hyperglycemic crisis. In the absence of unequivocal hyperglycemia, criteria 1) to 2) should be confirmed by repeated testing.

**Figure 1:**
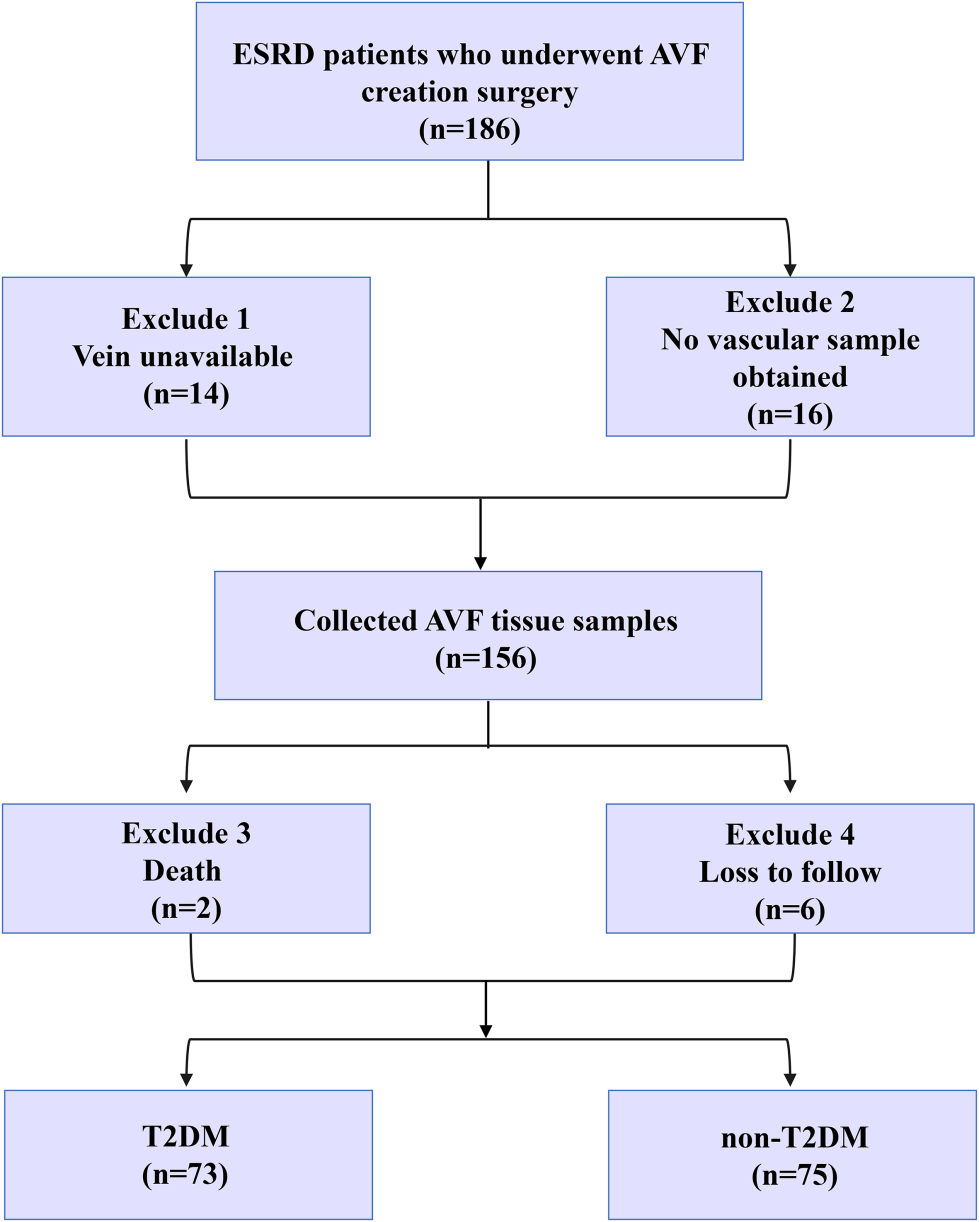
Tissue collection procedure of T2DM (n=73) and non-T2DM (n=75) groups. Initially, 186 patients with ESRD who underwent AVF surgery were enrolled in this study. Notably, 14 patients with unavailable veins and 16 cases with no vascular samples were excluded from this study. Next, 156 AVF tissue samples were collected. Two patients who died after surgery, and 6 patients lost to follow-up were also excluded. Finaly, 148 remaining patients were classified into the T2DM (n=73) and non-T2DM (n=75) groups. **Figure 1** illustrates the tissue collection procedure.

Initially, 186 patients with ESRD who underwent AVF surgery were enrolled in this study. Notably, 14 patients with unavailable veins and 16 cases with no vascular samples were excluded from this study. Next, 156 AVF tissue samples were collected. Two patients who died after surgery, and 6 patients lost to follow-up were also excluded. Finaly, 148 remaining patients were classified into the T2DM (n=73) and non-T2DM (n=75) groups. **Figure 1** illustrates the tissue collection procedure.

### 2.2 Tissue collection

Before AVF creation surgery, preoperative examination and evaluation of blood vessels were conducted for all participants, including a preoperative physical examination (assessment of upper limb blood pressure, arterial pulsation, upper limb oedema, and history of central or peripheral venous catheters) and a preoperative ultrasound examination. The blood pressure difference between the two upper limbs was required to be no greater than 20 mmHg, and normal pulsation was verified in the radial, ulnar, and brachial arteries of both upper limbs. Preoperative ultrasound assessments included measurements of the anastomotic artery diameter, venous bundle arm diameter, and venous non-bundle arm diameter.

All procedures were performed under local anesthesia, and 5–10 mm of non-utilized veins at the ligation site were collected during AVF creation surgery. Half of each collected AVF tissue sample was frozen in liquid nitrogen and stored at -80°C. The other half was fixed in 4% paraformaldehyde for subsequent histological staining.

### 2.3 DIA proteomic analysis

Following three washes with phosphate-buffered saline (PBS), the collected samples were minced and lysed in a lysis buffer (8 M urea, 100 mM Tris hydrochloride, pH 8.0) containing protease and phosphatase inhibitors, then sonicated for 1 min. The lysate was centrifuged at 14000 ×*g* for 10 min, and the supernatants were collected to determine protein concentrations using the Bradford assay (15). Proteins (20 µg) were loaded onto sodium dodecyl–polyacrylamide gel electrophoresis gels. Protein bands were visualized using Coomassie Blue R-250 staining. For the filter-aided sample preparation (FASP digestion) procedure, 200 µg of protein was reduced with 10 mM dithiothreitol at 56 °C for 30 min and alkylated with 10 mM iodoacetamide at room temperature in the dark for 30 min. Subsequently, 200 μg of protein was added to the filter unit with 200 μL of 8 M urea bicarbonate, then vortexed and centrifuged at 14000 ×*g* for 20 min. Finally, the protein suspensions were digested with 4 μg trypsin using a filter-aided method (Promega, Beijing, China) in 100 μL of 50 mM NH_4_HCO_3_ buffer overnight at 37 °C. The resulting peptides were collected as filtrates and then vacuum-dried using a Concentrator Plus (Eppendorf).

For the proteome profiling samples, peptides were analyzed on a timsTOF Pro mass spectrometer (Bruker) coupled with a high-performance liquid chromatography system (nanoElute 2, Bruker). Dried peptide samples re-dissolved in Solvent A (0.1% formic acid in water) were loaded onto a 2-cm self-packed trap column (100 μm inner diameter, 3 μm ReproSil-Pur C18-AQ beads, Dr Maisch GmbH) using Solvent A and separated on a 150 μm-inner-diameter column with a length of 15 cm (1.9 μm ReproSil-Pur C18-AQ beads, Dr Maisch GmbH) over a 75 min gradient (Solvent A: 0.1% Formic acid in water; Solvent B: 0.1% Formic acid in 80% ACN) at a constant flow rate of 600 nL/min (0–75 min, 0 min, 4% B; 0–10 min, 4-15% B; 10–60 min, 15-30% B; 60–69 min, 30-50% B; 69–70 min, 50-100% B; 70–75 min, 100% B). After separation by chromatography, the samples were analyzed using a timsTOF Pro 2 mass spectrometer. The detection mode was positive ionization, with the ion source voltage set to 1.6 kV. Both MS and MS/MS analyses were performed using time-of-flight (TOF) detection. The mass spectrometry scan range was set from 100 to 1700 m/z. The 1/K0 Start was set to 0.85 Vs/cm^2^, and the 1/K0 End was also set to 0.85 Vs/cm^2^. The ion accumulation and Rapm times were both set to 100 ms. Data acquisition was conducted in Parallel Accumulation Serial Fragmentation (PASEF) mode, with four PASEF mode fragment ion collections following each primary MS spectrum acquisition. A cycle window time of 0.53 seconds was employed. Secondary spectra within the charge range of 0–5 were obtained, with a dynamic exclusion time of 24 seconds set for the tandem mass spectrometry scans to avoid redundant scanning of fragment ions. The original data of mass spectrometry analysis were.d files, and MaxQuant was used for qualitative and quantitative analysis.

The overlapping targets were subjected to topological analysis using STRING (version 12.0) to construct a protein-protein interaction (PPI) network. All active interaction sources were selected, including text mining, experiments, databases, co-expression, neighborhood, gene fusion, and co-occurrence. A medium confidence combined score threshold of > 0.4 was applied to include meaningful interactions while filtering out non-specific associations.

### 2.4 Immunohistochemical staining

Following dewaxing using xylene, rehydration using ethanol, and elimination of endogenous peroxidase activity using 3% H_2_O_2_, AVF tissue sections were incubated with 0.5% Triton X-100 for 10 min and blocked with goat serum for 20 min. Tissue sections were then incubated overnight with primary antibodies against GPX4 (1:1000, DF6701, Affinity, Jiangsu, China), xCT (1:1000, DF12509, Affinity), α-SMA (1:1000, AF1032, Affinity), TGF-β (1:1000, AF1027, Affinity), IL-6 (1:1000, DF6087, Affinity), and TNF-α (1:500, AF7014, Affinity) at 4 °C. The following day, sections were incubated with a goat anti-rabbit secondary antibody (PV6001, ZSGB-BIO, Beijing, China). After three washes with PBS, sections were stained with diaminobenzidene and hematoxylin. ImageJ software (version 5.0; National Institutes of Health) was used to analyze the mean integrated optical density (IOD), allowing for calculation of relative protein expression.

### 2.5 Enzyme linked immunosorbent assay

Human 4-hydroxynonenal (4-HNE; E-EL-0128, Elabscience, Wuhan, China), human glutathione (GSH; E-EL-0026, Elabscience), human malondialdehyde (MDA; E-EL-0060, Elabscience), human superoxide dismutase 1 (SOD1; CB12115-Hu, COIBO BIO, Shanghai, China), human glutathione peroxidase 4 (GPX4; EH8916, FineTest, Wuhan, China), human acetyl-CoA acyltransferase 1 (ACAA1; ELH-ACAA1, RayBiotech, Guangzhou, China), human phosphoglycerate dehydrogenase (PHGDH; CB15362-Hu, COIBO BIO), human protein disulfide isomerase family A member 4 (PDIA4; CSB-EL017722HU, CUSABIO, Wuhan, China), human thioredoxin domain containing 5 (TXNDC5; CSB-EL025379HU, CUSABIO) ELISA kits were used to measure corresponding factors in serum samples obtained from patients, following the manufacturer’s instructions.

The ferrous iron (Fe^2+^), ferric iron (Fe^3+^), and total iron in cephalic vein tissues were analyzed using Iron Assay Kit (ab83366, Abcam, Cambridge, UK).

### 2.6 Western blotting analysis

Fifty milligrams of tissue were homogenized to a fine powder in liquid nitrogen. Total protein was extracted using RIPA lysis buffer (R0020; Solarbio, Beijing, China). The protein samples were separated by SDS-PAGE and transferred onto a PVDF membrane. After blocking with 5% skim milk at room temperature for 1 h, the membrane was incubated overnight at 4°C with the following primary antibodies: anti-GPX4 (1:1000, 30388-1-AP, Proteintech, Wuhan, China), anti-SOD1 (1:5000, 10269-1-AP, Proteintech), anti-ACSL4 (1:1000, ab155282, Abcam), anti-PTGS2 (1:1000, 12375-1-AP, Proteintech), or anti-β-actin (1:20000, 66009-1-Ig, Proteintech). Subsequently, the membrane was incubated with horseradish peroxidase (HRP)-conjugated goat anti-rabbit (ZB2301; ZSGB-Bio, Beijing, China) or goat anti-mouse (ZB2305; ZSGB-Bio) secondary antibody at room temperature for 1 h. Protein bands were visualized using an ECL chemiluminescence kit (C05-07004; Bioss, Beijing, China) and quantified with ImageJ software.

### 2.7 Analysis of covariance

To control for potential confounding factors, ANCOVA was performed to compare the adjusted mean levels of GPX4, TXNDC5, SOD1, PHGDH, PDIA4, ACAA1 between the T2DM and non-T2DM groups, with parathyroid hormone (PTH) level and dialysis age included as covariates. All statistical analyses were conducted using IBM SPSS Statistics (version 24).

### 2.8 Statistical analysis

The sample size in this study was validated by a statistical power calculation using the power.t.test function from the R package “pwr”. Python 3.9, R (version 4.1.0), CytoScape software, and a 3D slicer were employed to analyze differences in baseline parameters and proteomic results across groups. Data were analyzed using Graphpad Prism software. Normally distributed data are presented as the mean ± SD, with an unpaired *t*-test used for statistical analysis. Non-normally distributed data are presented as the M (1/4, 3/4), with independent sample nonparametric tests used for statistical analysis. Statistical data are expressed as percentages (%), and the χ2 test was used for comparison between groups. Results of a *P*-value of less than 0.05 were considered statistically significant. For ANCOVA, a two-tailed *P*-value< 0.05 was considered statistically significant.

## 3 Results

### 3.1 Clinicopathologic characteristics of the study population

Based on the inclusion and exclusion criteria, a total of 186 ESRD patients who underwent AVF creation surgery were finally enrolled in this study. The tissue collection procedure is illustrated in **Figure 1**.

The enrolled patients were divided into T2DM (n = 73) and non-T2DM (n = 75) groups. The mean age of patients in T2DM and non-T2DM groups was 62.050 ± 11.812 and 60.030 ± 9.791 years, respectively. The overall AVF maturation rate in the T2DM group was significantly lower than in the non-T2DM group (45.2% vs. 73.3%, *P* < 0.001). Dialysis age (*t*/*χ*^2^ = -2.059, *P* = 0.042) and CRP levels (*t*/*χ*^2^ = -6.238, *P* < 0.001) were significantly higher in the T2DM group compared with the non-T2DM group. Conversely, maturation rate (*t*/*χ*^2^ = 11.003, *P* = 0.001), PTH (*t*/*χ*^2^ = 4.493, *P* < 0.001), and the internal diameter of the cephalic vein one month post-operation (*t*/*χ*^2^ = 5.851, *P* < 0.001) were significantly lower in the T2DM group. No significant differences were observed in preoperative venous diameter, radial artery diameter, body mass index, antiplatelet drug use, or blood examination indices (hemoglobin, albumin, cholesterol, and triacylglycerol) between the two groups (**Table 1**).

**Table 1:** Clinicopathologic characteristics of the study population.

Observational index	T2DM (n=73)	non-T2DM (n=75)	*t*/*χ*^2^	*P*-value
Age, (mean ± SD)	62.050±11.812	60.030±9.791	-1.138	0.257
SBP (mmHg)	146.630±15.877	146.400±17.905	-0.083	0.934
DBP (mmHg)	82.000±7.612	82.547±8.571	0.410	0.683
BMI (kg/m^2^)	24.971±5.157	24.783±5.702	-0.209	0.835
Sex, male (%)	34 (46.6%)	41 (54.7%)	0.672	0.472
Dialysis age (month)	5.918±8.616	3.680±3.515	-2.059	0.042*
Maturation rate (%)	33 (45.2%)	55 (73.3%)	11.003	0.001**
Hypertension (%)	60 (82.2%)	63 (84.0%)	0.005	0.941
Cerebrovascular diseases (%)	10 (13.7%)	13 (17.3%)	0.147	0.702
Coronary heart disease (%)	14 (19.2%)	17 (22.7%)	0.102	0.749
Antiplate drugs (%)	13 (17.8%)	16 (21.3%)	0.111	0.739
Smoking (%)	15 (20.5%)	18 (24.0%)	0.094	0.759
Hemoglobin (g/L)	103.082±13.882	101.480±17.097	-0.625	0.533
Platelet (/L)	216.507±51.425	209.080±69.793	-0.735	0.463
Albumin (g/L)	37.886±5.192	38.749±5.054	1.025	0.307
Blood glucose (mmol/L)	8.089±2.546	7.892±3.891	-0.364	0.717
Cholesterol (mmol/L)	4.185±0.971	4.078±1.249	-0.582	0.561
Triglyceride (mmol/L)	2.157±1.074	2.226±0.992	0.405	0.686
Blood calcium (mmol/L)	2.294±0.234	2.366±0.227	1.900	0.059
Blood phosphorus (mmol/L)	1.769±0.446	1.729±0.445	-0.553	0.581
PTH (pg/mL)	198.839±76.588	290.622±157.332	4.493	<0.001***
Serum creatinine (μg/L)	628.137±222.252	633.387±192.357	0.154	0.878
Urea nitrogen (mg/dL)	22.531±8.528	21.987±7.222	-0.419	0.676
D-dimer (mg/L)	0.539±0.417	0.571±0.458	0.443	0.659
CRP (mg/L)	18.389±17.934	4.721±5.439	-6.238	<0.001***
Blood flow 1-month post-surgery (mL/min)	623.264±239.999	630.547±155.406	0.220	0.826
Cephalic vein ID 1-month post-surgery (mm)	4.381±0.626	5.209±1.049	5.851	<0.001***
Anastomotic stoma 1-month post-surgery (mm)	3.893±0.978	3.975±0.979	0.506	0.613
Radial artery 1-month post-surgery (mm)	3.333±0.650	3.387±0.715	0.478	0.633
Brachial artery 1-month post-surgery (mm)	5.044±0.780	5.115±0.710	0.578	0.564
PCVD, mm (unbunched arm)	2.346±0.407	2.334±0.473	-0.175	0.861
PCVD, mm (bunched arm)	2.937±0.421	3.045±0.573	1.308	0.193
Preoperative radial artery diameter (mm)	2.238±0.552	2.376±0.915	1.106	0.270

SBP, systolic blood pressure; DBP, diastolic blood pressure; BMI, body mass index; PTH, parathyroid hormone; CRP, C-reactive protein; ID, internal diameter; PCVD, preoperative cephalic vein diameter.

### 3.2 Proteomics analysis of differential expressed proteins in ESRD patients with T2DM

**Figure 2:**
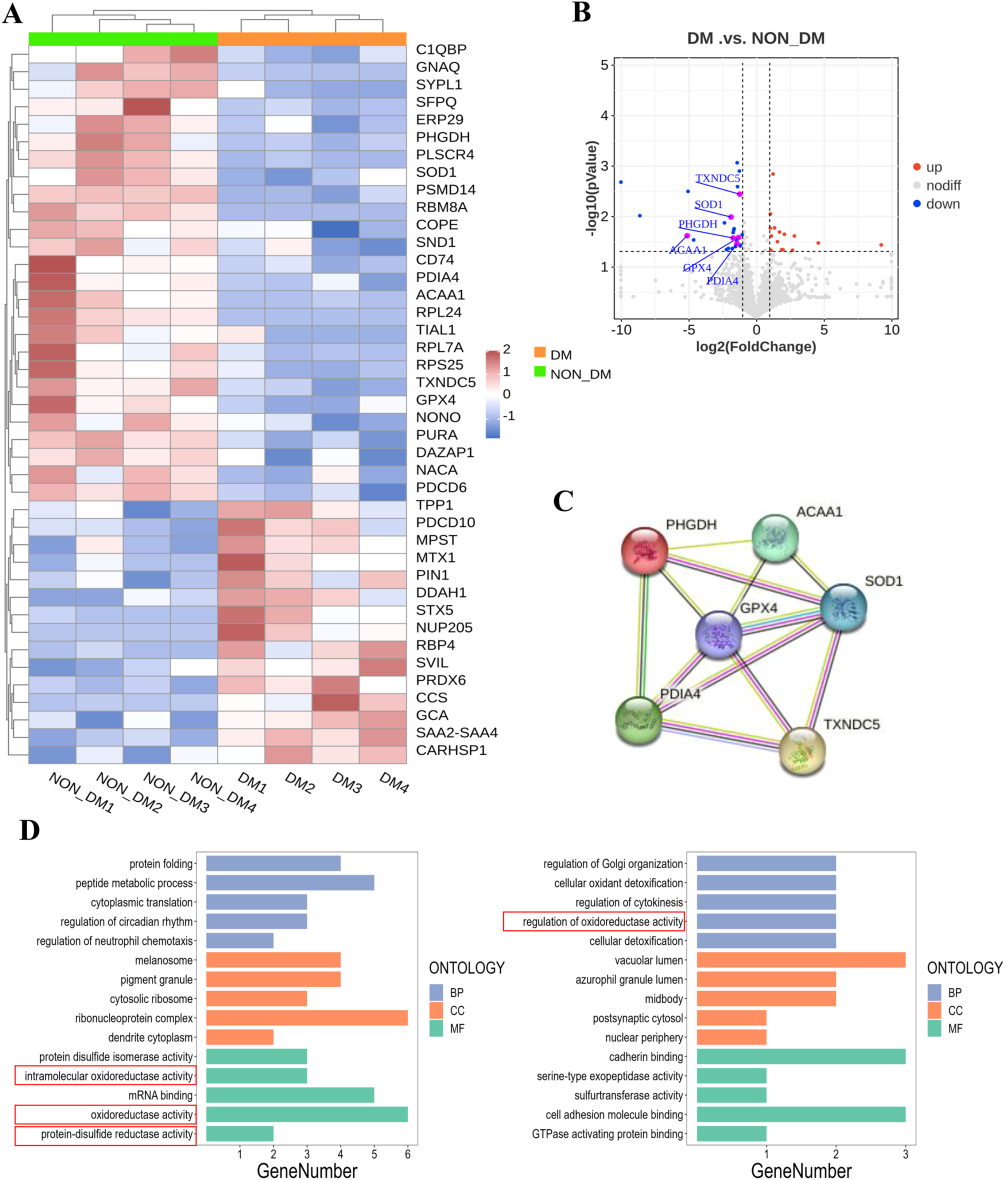
Proteomics analysis of differential expressed proteins in ESRD patients with T2DM. **(A)** Hierarchical clustering and heat map showing the differentially expressed proteins between T2DM and non-T2DM groups. Red and blue indicate up- and downregulation, respectively; **(B)** Volcano plot of the differentially expressed proteins; **(C)** PPI network of the differently expressed proteins in non-T2DM group; **(D)** GO analytical results. n=4.

The hierarchical clustering in **Figure 2A** illustrates the differences in protein expression between the T2DM and non-T2DM groups. Compared with the non-T2DM group, 26 proteins were upregulated and 15 were downregulated in the T2DM group (**Figure 2B**). A PPI network illustrated the interactions between enriched proteins identified using GO analysis, including TXNDC5, SOD1, GPX4, PHGDH, PDIA4, and ACAA1 (**Figure 2C**, **Table 2**). We further analyzed these differentially expressed proteins using Gene Ontology (GO) analysis, which revealed that these proteins were enriched in intramolecular oxidoreductase, oxidoreductase, and protein-disulfide reductase activities (**Figure 2D**).

**Table 2:** The expression changes of proteins in the regulation of oxidoreductase activity.

ID number	Protein	FC	Log2FC	*P*-value
2879	GPX4	0.405676552	-1.302	0.026*
81567	TXNDC5	0.430592838	-1.216	0.004**
6647	SOD1	0.275476269	-1.860	0.010**
26227	PHGDH	0.309613837	-1.691	0.027*
9601	PDIA4	0.374192605	-1.418	0.035*
30	ACAA1	0.029160422	-5.100	0.024*

GPX4, glutathione peroxidase 4; TXNDC5, thioredoxin domain containing 5; SOD1, superoxide dismutase 1; PHGDH, phosphoglycerate dehydrogenase; PDIA4, protein disulfide isomerase family A member 4; ACAA1, acetyl-CoA acyltransferase 1.

In addition, ANCOVA was performed to detect the expression of those proteins between non-T2DM and T2DM groups with adjustment for PTH and dialysis age. Data in **Table 3** showed that, the adjusted mean level of SOD1 was significantly lower in the T2DM group (1928.861 ± 83.157 pg/mL) than in the non-T2DM group (1928.861 ± 83.157 pg/mL), with a mean difference of -427.158 pg/mL (95% CI: -666.585 to 187.73; *F*(1, 144) = 12.435, *P* = 0.001, partial η² = 0.079). Similarly, the adjusted mean level of GPX4 was significantly lower in the T2DM group (1431.371 ± 96.743 pg/mL) compared to the non-T2DM group (3166.907 ± 95.346 pg/mL), with a mean difference of -1735.536 pg/mL (95% CI: -2014.083 to 1456.990; *F*(1, 144) = 151.670, *P* < 0.001, partial η² = 0.513). In contrast, no statistically significant differences between the groups were observed for the adjusted mean levels of ACAA1, PHGDH, PDIA4, or TXNDC5.

**Table 3:** ANCOVA of differentially expressed proteins between non-T2DM and T2DM groups, with adjustment for PTH and dialysis age.

Protein	Non-T2DM (adj.Mean ± SE)	T2DM (adj.Mean ± SE)	Difference (95% CI)	F (1, 144)	*P*-value	Partial η²
SOD1 (pg/mL)	2356.019 ± 81.956	1928.861 ± 83.157	-427.158 (-666.585-187.73)	12.435	0.001**	0.079
GPX4 (pg/mL)	3166.907 ± 95.346	1431.371 ± 96.743	-1735.536 (-2014.083-1456.990)	151.670	<0.001***	0.513
ACAA1 (pg/mL)	57.491 ± 3.670	66.355 ± 3.724	8.864 (-1.858-19.586)	2.670	0.104	0.018
PHGDH (ng/mL)	46.298 ± 2.292	50.893 ± 2.325	4.595 (-2.100-11.290)	1.840	0.177	0.013
PDIA4 (ng/mL)	909.699 ± 38.031	878.054 ± 38.588	-31.645 (-142.75-79.459)	0.317	0.574	0.002
TXNDC5 (ng/mL)	7.140 ± 0.360	7.728 ± 0.366	0.587 (-0.465-1.640)	1.217	0.272	0.008

ANCOVA, analysis of covariance; PTH, parathyroid hormone; GPX4, glutathione peroxidase 4; TXNDC5, thioredoxin domain containing 5; SOD1, superoxide dismutase 1; PHGDH, phosphoglycerate dehydrogenase; PDIA4, protein disulfide isomerase family A member 4; ACAA1, acetyl-CoA acyltransferase 1.

### 3.3 Immunohistochemical staining for confirming the expression changes of oxidoreductase activity-related proteins

**Figure 3:**
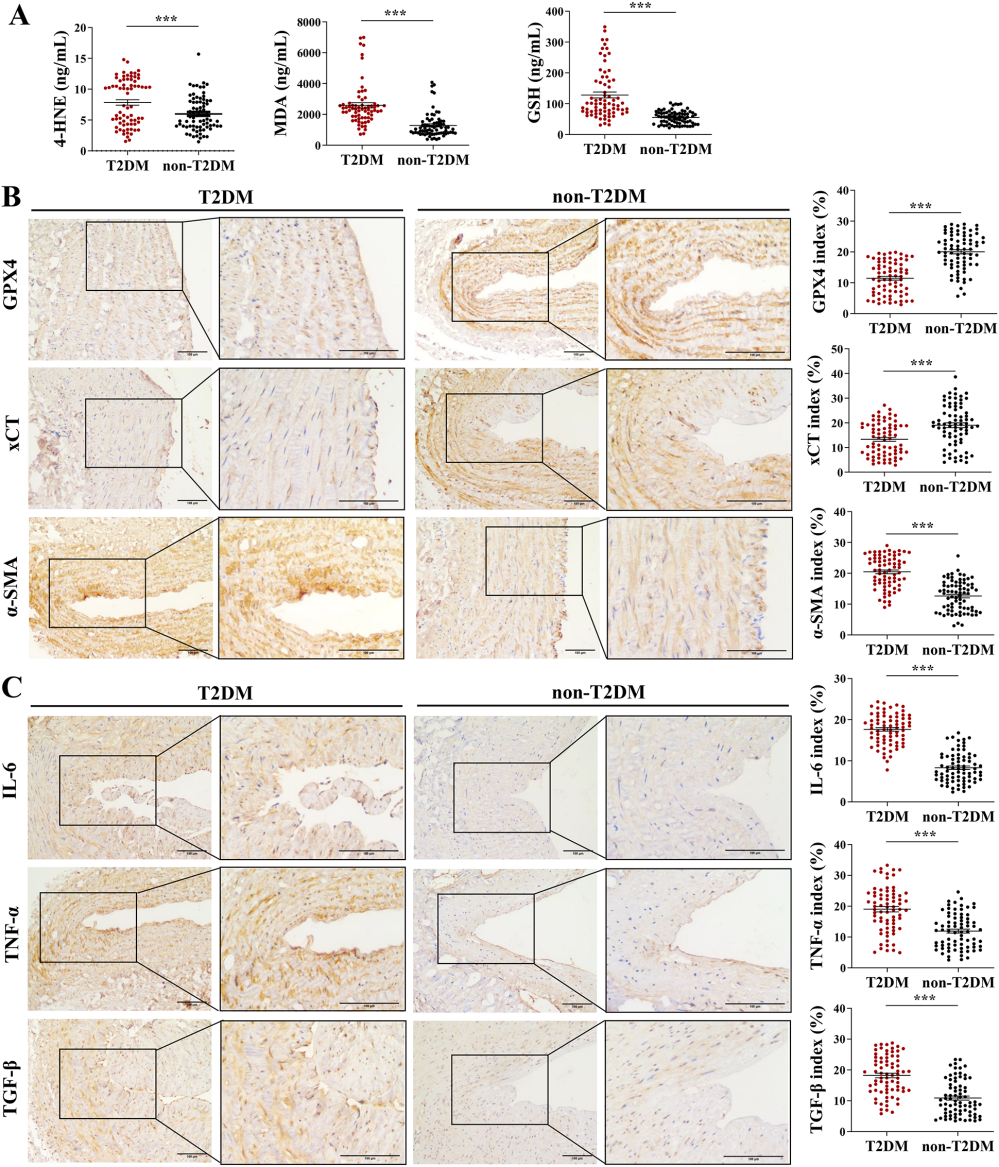
Immunohistochemical staining for confirming the expression changes of oxidoreductase activity-related proteins. **(A)** ELISA for detection of serum levels of 4-HNE, MDA, and GSH of T2DM (n=73) and non-T2DM (n=75) groups; **(B, C)** Immunohistochemical staining of AVF tissue samples collected from ESRD patients with T2DM (n=73) or non-T2DM (n=75). The mean integrated optical density (IOD of GPX4, xCT, α-SMA, IL-6, TNF-α, and TGF-β was calculated using ImageJ software to calculate relative protein expression. Scale bar = 100 μm. Data are presented as the mean ± SD, with an unpaired *t*-test used for statistical analysis. ***P < 0.001.

Serum samples collected from T2DM and non-T2DM patients were analyzed to determine the concentrations of oxidoreductase activity-related proteins. As shown in **Figure 3A**, the serum concentrations of 4-HNE, MDA, and GSH were significantly higher in the T2DM group compared with the non-T2DM group. Immunohistochemical staining results demonstrated that the index of GPX4 and xCT was lower, while the indices of α-SMA, IL-6, TNF-α, and TGF-β were higher in the T2DM group than in the non-T2DM group (**Figures 3B, C**).

### 3.4 Confirmation of the dysregulation of ferroptosis-related proteins

**Figure 4:**
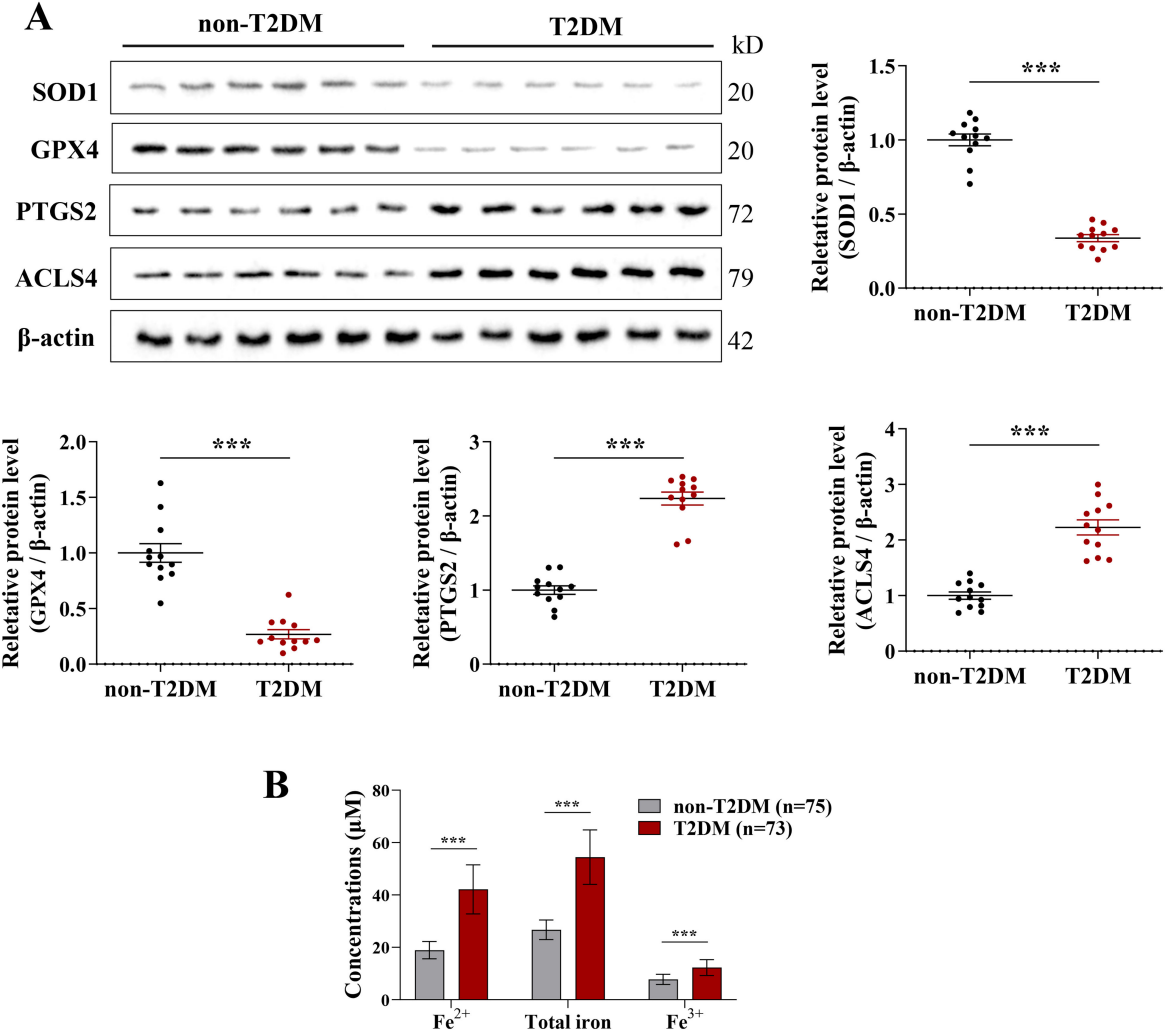
Confirmation of the expression of ferroptosis-related proteins. **(A)** Western blotting analysis for detection of SOD1, GPX4, PTGS2, and ACLS4 expression in cephalic vein tissues of T2DM (n=12) and non-T2DM (n=12) groups. **(B)** ELISA for detection of Fe^2+^, total iron, and Fe^3+^ levels in cephalic vein tissues of T2DM (n=73) and non-T2DM (n=75) groups. Data are presented as the mean ± SD, with an unpaired *t*-test used for statistical analysis. ***P < 0.001.

Subsequently, we collected cephalic vein tissues from patients in both groups and performed Western blot analysis to examine the expression of ferroptosis-related proteins. The results revealed that, compared with the non-T2DM group, the expression levels of SOD1 and GPX4 were significantly decreased in the T2DM group, whereas the expression of PTGS2 and ACSL4 was markedly increased (**Figure 4A**). Furthermore, ELISA results demonstrated that the levels of Fe²^+^, total iron, and Fe^3^^+^ were significantly elevated in the T2DM group relative to the non-T2DM group (**Figure 4B**). These findings indicate abnormal expression of ferroptosis-related proteins in the cephalic vein tissues of patients with ESRD combined with T2DM.

## 4 Discussion

Proteins serve as direct effectors in the biological processes of human diseases and are differentially expressed in response to therapeutic interventions. Proteomics is an effective technology for detecting changes in protein expression in cells, tissues, organs, and organisms. Proteomic technologies monitor changes in protein expression during disease progression and identify potential biomarkers for diagnosis and prognosis (16). In this study, we performed DIA proteomic analysis on AVF tissues collected from ESRD patients with or without T2DM. A total of 26 upregulated and 15 downregulated proteins were identified in the T2DM group compared with the non-T2DM group. Using ELISA, immunohistochemical staining, and Western blotting analysis, proteins enriched in the regulation of oxidoreductase activity (including ferroptosis-related proteins) were confirmed to be differentially expressed in T2DM patients.

As the primary cause of ESRD, T2DM induces expression changes in a wide array of proteins (17). However, the regulatory mechanisms underlying these differentially expressed proteins in the progression of diabetic ESRD remain largely unknown. In this study, we conducted DIA proteomic analysis to examine the changes in protein expression in AVF tissues collected from ESRD patients with or without T2DM. The results showed that differentially expressed proteins were enriched in oxidoreductase and protein-disulfide reductase activities, highlighting the role of oxidoreductase activity in this disease. TXNDC5, PDIA4, GPX4, and SOD1 were among the most enriched proteins. TXNDC5, a member of the protein disulfide isomerase (PDI) family, contributes to regulating various biological processes (18). TXNDC5 is essential for protecting cells from oxidative stress (19) and is a valuable biomarker for diabetes mellitus (20). Another PDI family member, PDIA4, functions as a reductase, oxidase, and molecular chaperone in the endoplasmic reticulum (21). Notably, recent studies have reported that PDIA4 confers resistance to ferroptosis through the regulation of xCT in tumor cells (22). GPX4 and SOD1 are well-known antioxidant proteins, with their roles in regulating ferroptosis receiving considerable attention. A primary feature of ferroptosis is the iron-dependent oxidative destruction of the cell membrane following the failure of the cellular antioxidant system. During ferroptosis, levels of GPX4 and SOD1 significantly decrease (23). Additionally, ferroptosis is involved in the development of various diabetic complications, including diabetic kidney disease (24, 25). These findings suggest that oxidoreductase activity-related proteins (including ferroptosis-related proteins) may serve as potential biomarkers for ESRD combined with T2DM.

Our proteomics analytical results revealed an extreme downregulation of ACAA1 in diabetic vascular tissues (Log_2_FC: -5.100). ACAA1, a key enzyme in mitochondrial fatty acid β-oxidation, acts as a key regulator in fatty acid metabolism. Its profound deficit may indicate a severe compromise in local energy homeostasis, which affecting the diabetic pathology of metabolic inflexibility and impaired fatty acid utilization (26). Interestingly, when controlling for major confounding variables (PTH and dialysis age) via ANCOVA, the association between T2DM status and ACAA1 levels was attenuated. It seems that, the dramatic ACAA1 depletion may influenced by PTH and dialysis age, a possibility that requires further confirmation.

Considering the association of GPX4 and SOD1 with ferroptosis, we further analyzed changes in the expression of ferroptosis-related factors in ESRD patients with T2DM. Serum concentrations of 4-HNE, MDA, and GSH were significantly higher in ESRD patients with T2DM. Furthermore, GPX4, xCT, and GPX4 were downregulated while PTGS4 and ACLS4 were upregulated in AVF tissues from ESRD patients with T2DM. Our findings, along with previous reports (27, 28), suggesting a ferroptosis-related molecular signature in the progression of diabetic ESRD.

Diabetic kidney disease involves a chronic inflammatory response during disease progression (29). Oxidoreductase activity and inflammatory response is strongly associated. The inflammatory response is a physiological defense mechanism of the body against external stimuli that clears pathogens and necrotic tissues; however, excessive inflammatory responses can lead to oxidative stress (30). During inflammation, white blood cells release various oxidants, such as superoxide and hydroxyl free radicals. These oxidants attack cell membranes, nucleic acids, proteins, and other biomolecules, inducing oxidative stress and aggravating the inflammatory response (31). In this study, the expression of pro-inflammatory proteins, i.e., IL-6, TNF-α, and TGF-β, was higher in the AVF tissues of ESRD patients with T2DM, indicating local inflammation in AVF tissues associated with diabetes. However, further research is needed to determine whether inflammation- and oxidative stress-driven ferroptosis is associated with AVF maturation failure.

The alterations of ferroptosis-related proteins we observed in AVF tissues (including SOD1, GPX4, PTGS2, ACLS4, and iron levels) are local manifestations of the systemic metabolic derangements in ESRD-T2DM. The AVF tissue is a “victim” of this hostile systemic environment. While the changes are measured locally, their drivers are systemic. The pathophysiology of both ESRD and T2DM involves endothelial dysfunction, accelerated atherosclerosis, and impaired vascular remodeling (32, 33), all processes where the vasculature is the central player. The discovery of ferroptosis in AVF tissues directly links this novel cell death pathway to these established vascular pathologies. It suggests that ferroptosis may be a key mechanistic bridge between the systemic metabolic milieu and the devastating vascular complications in this patient population.

This study has several limitations that must be considered. The inherent selection bias of a cohort requiring AVF surgery means our findings are most relevant to this specific clinical context and not necessarily to the entire ESRD-T2DM population. Furthermore, despite observing a distinct proteomic signature, significant baseline differences in factors such as CRP and dialysis vintage introduce potential residual confounding; thus, the contributions of T2DM per se versus these comorbidities cannot be fully disentangled. Finally, the generalizability of results from the cephalic vein to other tissues or vascular territories remains uncertain. To address these points, a multi-faceted research trajectory is proposed. First, validation of the identified biomarkers will be pursued in a larger, independent cohort, utilizing longitudinal plasma samples to rigorously correlate expression levels with clinical outcomes. Second, future studies with prospectively matched cohorts are necessary to isolate the specific pathophysiological impact of diabetes. Finally, the functional implication of key targets like GPX4 and SOD1 will be experimentally defined through *in vitro* models incorporating genetic and pharmacological interventions to elucidate their causal roles in diabetic vascular remodeling.

## 5 Conclusion

We conducted DIA proteomic analysis to identify differentially expressed proteins between ESRD patients with and without T2DM. Proteins involved in the regulation of oxidoreductase activity were significantly differentially expressed in ESRD patients with T2DM. ELISA, immunohistochemical staining, and Western blotting confirmed that the ferroptosis-related proteins SOD1, GPX4 and xCT were downregulated, while PTGS2 and ACLS4 were upregulated in ESRD combined with T2DM.

## Data Availability

The datasets presented in this study can be found in online repositories. The names of the repository/repositories and accession number(s) can be found in the article/**Supplementary Material**.
